# Problem-solving training to improve caregiver burden and depressive symptoms among dementia caregivers: personal and clinical factors of responders vs. non-responders

**DOI:** 10.3389/fpubh.2025.1682373

**Published:** 2025-10-10

**Authors:** Shannon B. Juengst, Matthew Lee Smith, Kristin Wilmoth, Brittany Wright, Gang Han, Charlene Supnet, Gladys Maestre

**Affiliations:** ^1^Brain Injury Research Center, TIRR Memorial Hermann, Houston, TX, United States; ^2^Department of Physical Medicine & Rehabilitation, University of Texas Southwestern Medical Center, Dallas, TX, United States; ^3^Center for Community Health and Aging, Texas A&M University, College Station, TX, United States; ^4^Center for Health Behavior, School of Public Health, Texas A&M University, College Station, TX, United States; ^5^Department of Psychiatry, University of Texas Southwestern Medical Center, Dallas, TX, United States; ^6^Department of Epidemiology and Biostatistics, School of Public Health, Texas A&M University, College Station, TX, United States; ^7^Department of Neurology, School of Medicine, Washington University in St. Louis, St. Louis, MO, United States; ^8^School of Medicine, One West University Boulevard, University of Texas Rio Grande Valley, Brownsville, TX, United States

**Keywords:** caregiver, dementia, Alzheimer’s disease and related dementia, problem-solving, Spanish language, dementia care, psychosocial intervention, metacognitive strategies

## Abstract

**Introduction:**

Metacognitive strategy training interventions, like Problem-Solving Training/Descubriendo Soluciones Juntos (PST/DSJ), have efficacy for improving caregiver burden and depressive symptoms. We previously demonstrated that PST/DSJ improved caregiver burden and depressive symptoms among caregivers of adults with Alzheimer’s Disease and related dementias (ADRD), regardless of the number of sessions or boosters received. However, these results did not examine factors characterizing those who responded (improvement in caregiver burden or depressive symptoms) or did not respond to the intervention.

**Objective:**

To identify key personal and clinical factors associated with response to PST/DSJ. Personal factors included age, gender, race, Hispanic ethnicity, education, and employment status. Clinical factors included care recipient diagnosis and dementia severity, caregiver problem-solving skills at baseline, caregiving experiences (caregiver life social support, satisfaction and resentment with the caregiving role, anger toward the care recipient, and care recipient aggressive, depressive, and forgetful behaviors), and social disconnection, caregiver burden, and depressive symptoms.

**Method:**

We conducted a 2 × 2 randomized controlled optimization trial to test remotely delivered PST/DSJ to ADRD caregivers (NCT04748666). Primary outcomes were caregiver burden, measured by the Zarit Burden Interview (ZBI), and depressive symptoms, measured by the Patient Health Questionnaire-8 (PHQ-8). Response to PST/DSJ was defined for each primary outcome as a clinically important change (defined as ≥1 point on ZBI and ≥3 on PHQ) from baseline to 6-month follow-up.

**Results:**

Ninety-one caregivers were included in responder analysis, with 55 (60.4%) demonstrating a clinically meaningful improvement in caregiver burden and/or depressive symptoms. No personal factors were associated with being a Responder (vs. Non-Responder). Clinical factors associated with being a Responder were greater care recipient dementia severity (FAST score, *p* < 0.01), lower baseline caregiver life satisfaction (*p* = 0.05), higher baseline caregiver overload (*p* = 0.05), higher baseline caregiver burden (*p* = 0.01), and more baseline depressive symptoms (*p* < 0.01).

**Conclusion:**

Most caregivers demonstrated a clinically meaningful improvement in caregiver burden and/or depressive symptoms after receiving PST/DSJ. Notably, those who responded had higher symptoms of distress, including caregiver burden and overload and depressive symptoms and lower life satisfaction, and had care recipients with more severe dementia, indicating that those benefiting from the intervention were those most in need of this support.

**Clinical trial registration:**

ClinicalTrials.gov, identifier is NCT04748666.

## Introduction

Alzheimer’s disease or related dementias (ADRD), including vascular dementia, Lewy Body dementia, and other dementias, represent a substantial public and personal health burden. Informal caregivers—typically spouses, adult children, or other family members – provide day-to-day support to individuals with ADRD, totaling approximately 18.4 billion hours of unpaid care per year (based on 2023 data) with an estimated value of $346.6 billion (1–4). Over 11 million individuals are currently informal caregivers of a person with ADRD (4). Being an informal caregiver to someone with ADRD can lead to depression, health problems, increased alcohol use, caregiver burden, and poorer quality of life (5–10). Caregiver distress also affects the health and well-being of care recipients (11), with caregiver burden emerging as a direct predictor of institutionalization and of care recipient behavioral and psychological symptoms (11). Moreover, caregiver burden is substantial in the underserved US Hispanic/Latino population, with few available linguistically and culturally appropriate resources (12) despite the higher likelihood of developing dementia for older Hispanic/Latino adults (4, 13).

To date, most interventions for dementia caregivers are primarily focused on providing them with education about ADRD and providing support to manage the needs and behaviors of their care-recipient (14–16). There is growing evidence that interventions focused on emotional support and stress management for caregivers may help in reducing or managing caregiver burden (17, 18). This highlights the importance of evidence-based interventions to support caregivers in managing their own needs and stressors, including ones focused on enhancing problem-solving skills, rather than just the needs of their care-recipients (16).

Even when interventions are shown to improve caregiver outcomes, knowing who is most likely to benefit from these interventions and who may need additional support or intervention adaptations to benefits remains unclear. Very few studies identify factors that contribute to response (i.e., meaningful improvement in outcomes) vs. non-response after caregiver support interventions. One study evaluating factors affecting change in depressive symptoms after a stroke caregiver intervention found that responders generally had a more active coping style and were less reliant on the counseling relationship (19), suggesting the importance of intervention components that promote self-efficacy and self-management. Further, non-responders more frequently endorsed a history of psychologic disorder and had higher levels of anger compared to responders (19), so those with more psychological distress (and arguably in most need of support) may be less likely to benefit. A qualitative study that surveyed non-responders and interventionists about a caregiver support intervention identified specific supports to meet the needs of non-responders: providing more support specific to caregiving, spending more time processing the caregiver’s emotions, providing skills and psychoeducation materials based on the caregiver’s needs, and working with caregivers to identify ways they can ask for help or strengthen interpersonal relationships (20).

Problem-Solving Training (PST)/Descubriendo Soluciones Juntos (DSJ) is an evidence-based, bilingual strategy training intervention that promotes proactive coping skills and self-efficacy by teaching a simple, systematic approach to problem-solving, including thorough problem assessment, generating and selecting solutions for specific self-identified goals, developing detailed plans of action, and evaluating and adapting plans as needed to support goal achievement (21–33). PST has been translated and culturally adapted for Spanish-speaking caregivers (DSJ) (34) and caregivers can receive the intervention via telephone or videoconference (23, 35, 36), circumventing many known barriers to caregiver support. PST has demonstrated efficacy in improving caregiver burden and reducing mood symptoms (22, 24, 27–30, 37) and negative problem-solving orientation (38, 39). We previously demonstrated that PST/DSJ led to improvements in caregiver burden and depressive symptoms among caregivers of adults with ADRD, regardless of the number of sessions or boosters received (39). However, these results examined participants in aggregate and did not examine factors characterizing those who responded or did not respond to the intervention, which is important to understand for personalizing intervention approaches and providing the best support to all who need it.

The objective of this study was to identify key personal and clinical factors associated with response to PST/DSJ, defined as improvement in caregiver burden and/or improvement in depressive symptoms, among ADRD caregivers. Personal factors included age, gender, race, Hispanic ethnicity, education, and employment status. Clinical factors included care recipient diagnosis and dementia severity, caregiver social problem-solving skills at baseline (pre-intervention), caregiving experiences, and baseline caregiver burden and depressive symptoms.

## Materials and methods

### Design

The CaDeS study was a 2×2 factorial design randomized controlled trial to test differential effects of number of sessions and booster sessions of PST/DSJ on caregiver burden and depressive symptoms among English- and Spanish-speaking caregivers of adults with ADRD (NCT04748666). Details about the study design and methods are provided in the published study protocol (40) and in the primary outcomes paper (39). Participants were randomized to receive 3 or 6 sessions with or without booster sessions. As reported in the published results for the primary trial aim, we found a main effect of time (improvement in both caregiver burden and depressive symptoms from baseline to 6-months post-baseline) with no significant difference for number of sessions or presence of booster sessions (39). Therefore, for the aim of this study to examine differences in those who did and did not respond to PST/DJS, we pooled all participants across study arms and categorized them based on improvement in the two primary outcomes, regardless of group assignment.

### Participants

Participants (*n* = 91) were informal caregivers of persons with ADRD. Inclusion criteria were that the participant identified as a caregiver (i.e., a family member, spouse/partner, or friend) with more than a 1-year relationship with the care recipient, spoke English or Spanish, was over 18 years old and able to self-consent, and endorsed some depressive symptoms and/or caregiver burden symptoms (scoring ≥2 on the PHQ-2 and/or ZBI-4). The PHQ-2 assesses the two hallmark symptoms of depression (41), with scores ranging from 0 to 6 and a score ≥2 validated as a cut-off for potential depression. The ZBI-4 is a short screener for caregiver burden with a score of ≥2 validated as a cut-off for notable caregiver burden (42).

We determined that a sample size of 26 per arm (*n* = 104 total) would achieve 80% power at a significance level of 0.05 to detect the improvements between any two arms of 30% vs. 65, 40% vs. 75, 50% vs. 83, and 60% vs. 90%, accounting for 10% attrition (40). We consented *n* = 106 participants, but randomized *n* = 104 (2 withdrawn prior to randomization), and 7 (6.7%) were lost prior to the intervention beginning. Of the *n* = 97 who started the intervention, 6 were lost to follow-up by the 6-month assessment (6.2%), leaving us with *n* = 91 participants to include in responder analysis. The percentage of participants who completed 100% of sessions ranged from 82.1 to 95.2% across study arms.

### Intervention

PST/DSJ teaches individuals a simple step-by-step process, to solve problems and achieve goals. A trained coach teaches participants the PST/DSJ strategy and then guides them through iterative practice applying it to goals of their choosing (34, 39, 40, 43). The strategy employs an easy to remember mnemonic: A = Assess the problem/ A = Analice el problema; B = Brainstorm solutions/ B = Buscar soluciones; C = Consider solutions and Choose one/ C = Considere y escoja; D = Develop a plan and Do it/ D=Desarrollar un plan y ¡Desempeñelo!; E = Evaluate/E = Evaluar y Evolucionar; F = Flex. Sessions were conducted by telephone or Microsoft Teams by Coaches with master’s level training. Detailed description of the 3- and 6-session versions of PST/DSJ are provided in the published protocol. Briefly, both versions included training participants how to use the ABCDEF strategy, with the six session version allowing more sessions for coach-supported iterative practice applying the strategy (26). During booster sessions, which occurred monthly for 6 months if assigned, participants followed up with their coach about progress they had made using the strategy, received extra supported practice, and discussed opportunities for using PST/DSJ in the future. Intervention fidelity, assessed using our established fidelity protocol (27), was excellent at 95%.

### Outcome measures

We collected:

1. Demographic data: age, gender, race, Hispanic ethnicity, education (≤ High School vs. > High school), and employment status (full-time or part-time vs. retired or unemployed).
2. Caregiver personal factors: social problem-solving skills [Social Problem-Solving Skills Inventory (44)] and social disconnectedness [Upstream Social Interaction Risk Scale (45, 46)].
3. Caregiving-related information: care recipient diagnosis (Alzheimer’s disease vs. Other), care recipient dementia severity [Functional Assessment Staging Tool (47) for dementia score], and family caregiving experiences (48), comprising caregiver life satisfaction, social support, overload, satisfaction and resentment with the caregiving role, anger toward the care recipient, help needed by and provided to the care recipient, and care recipient aggressive, depressive, and forgetful behaviors.
4. Clinical outcome measures: caregiver burden [Zarit Burden Interview (49, 50)] and depressive symptoms [Patient Health Questionnaire (41, 51)].

Primary outcomes were caregiver burden, measured by the Zarit Burden Interview (ZBI), and depressive symptoms, measured by the Patient Health Questionnaire-8 item version (PHQ-8). The ZBI (49, 50) consists of 22 items and measures self-reported caregiver burden with included items covering overall well-being, social and family life, finances, perceived control, and emotional health. ZBI scores range from 0 to 88. The PHQ (41, 51) is a depression screening tool based on the DSIM-IV-TR symptoms of a major depressive episode. Scores range from 0 to 24 for the 8-item version.

Response to the intervention was defined for each primary outcome as a clinically important change from baseline to 6-month follow-up (final follow-up, 3 months post-end of intervention). For ZBI, we used a distribution-based method (52), defining Responders as those who improved by ≥1 point, equivalent to 1 standard error of the mean (SEM) of the sample (53) vs. Non-responders who did not. For PHQ, we based our definition of response on consensus-methods (52), which ranged from 2 to 3 points. We defined Responders as those who improved by ≥3 points (54) vs. Non-responders who did not.

### Statistical analysis plan

We calculated the percentage of responders for each outcome and overall (Responder for Caregiver Burden AND/OR Depressive symptoms vs. Non-responder for both). We first descriptively present differences between Responders and Non-Responders on all covariates. Next, we conducted bivariate analyses, including Mann–Whitney U tests and Chi-squared tests, to determine statistically significant differences between Responders and Non-Responders for all covariates. We used the overall Responder variable (improvement in either outcome) as our primary indicator of response to intervention, as inclusion criteria for the study were a positive screen for caregiver burden OR depressive symptoms (ZBI-4 or PHQ-2 scores) rather than both. A *p*-value of ≤0.05 was deemed statistically significant, and all tests were two-sided.

### Ethics statement

All research procedures were in accordance with the Declaration of Helsinki, and all participants provided informed consent. The UT Southwestern Medical Center’s Institutional Review Board (IRB) served as the single IRB for the study, with other sites as reliance sites. Protocols were established for managing any crises that arose in the context of intervention delivery, and there were no serious adverse events. This trial is registered to ClinicalTrials.gov Identifier: NCT04748666.

## Results

Ninety-one participants completed the 6-month follow-up assessment (88% retention of those who consented) and were included in these analyses (39). Based on initial screening, all participants (100%) met the criterion cut-off on the ZBI-4, and 27 participants (29.7%) met the criterion cut-off on the PHQ-2. The mean change in ZBI for all participants was −3.0 (SD = 9.2, Cohen’s d = 0.33) and for PHQ was −1.1 (SD = 3.6, Cohen’s d = 0.31).

**Figure 1:**
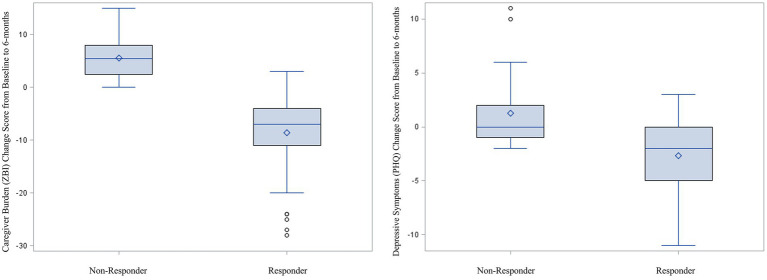
Boxplots comparing change in caregiver burden and depressive symptoms between responders and non-responders; positive scores indicate worsening symptoms; negative scores indicate improving symptoms.

For caregiver burden, there were 51 (56.0%) participants who improved by 6-month follow-up and 40 (44.0%) who did not improve. For depressive symptoms, there were 27 (29.7%) who improved and 64 (70.3%) who did not improve. Of these 64 who did not improve on the PHQ-8, almost all had stable depressive symptoms, with many not meeting the initial screening criteria for depressive symptoms (i.e., they did not have meaningful depressive symptoms to improve). Close to two-thirds of the sample (60.4%, *n* = 55) showed meaningful improvement on at least one of the outcomes (Responders), with a little over one-third of the sample (39.6%) not showing meaningful improvement for either caregiver burden or depressive symptoms (Non-Responders). Table 1 shows the cross tabulation of Responders and Non-Responders across both outcomes. Notably, among those whose depressive symptom scores improved, only 4 (14.8%) did not also improve for caregiver burden. Figure 1 shows the distribution of change scores for the ZBI and PHQ for Responders and Non-Responders.

**Table 1:** Cross tabulation of responders and non-responders for caregiver burden and depressive symptoms.

Caregiver burden	Depressive symptoms		Total
	Responder	Non-responder	
Responder	23	28	51
Non-responder	4	36	40
Total	27	64	91

*Responder is defined as a decrease in the outcome (≥1 point on the Zarit Burden Interview for caregiver burden; ≥3 points on the Patient Health Questionnaire for depressive symptoms) from baseline to second follow-up (6 months post-intervention). All 91 participants screened positive at baseline for caregiver burden, whereas only 27 (29.7%) screened positive for depressive symptoms (59 did not).

Table 2 presents personal and clinical characteristics for all participants (*n* = 91) and descriptive and bivariate analyses between the combined Responder vs. Non-Responder groups. Statistically significant differences between groups for bivariate analyses were observed, with Responders reporting lower Caregiver Life Satisfaction (*p* = 0.05) and more feelings of Caregiver Overload (*p* = 0.05), more Caregiver Burden (*p* = 0.01), and greater Depressive Symptoms (*p* < 0.001) at baseline compared to Non-Responders. Responders had care recipients with higher FAST scores (dementia severity, *p* < 0.01) compared to their Non-Responder counterparts, with respective median scores of 6 (IQR = 5.7) indicating moderately severe dementia, vs. 5 (IQR = 4.6), indicating midstage dementia (see Table 2).

**Table 2:** Differences in personal and clinical characteristics between responders and non-responders to problem-solving training for either caregiver burden or depressive symptoms.

	All	Responders^±^	Non-responders	*p*-value
	*N* = 91	*N* = 55	*N* = 36	
A. Personal factors				
Age years (median [IQR])	61, [52, 72]	59, [52, 71]	65.5 [52, 73]	0.6
Gender				0.56
CIS-Female	77 (86%)	48 (87%)	29 (83%)	
CIS-Male	13 (14%)	7 (13%)	6 (17%)	
Employment				0.12
Yes (Full-time or Part-time)	42 (46%)	29 (53%)	13 (36%)	
No (Retired or Unemployed)	49 (54%)	26 (47%)	23 (64%)	
Diagnosis of care recipient				0.16
Alzheimer’s Disease	56 (62%)	37 (67%)	19 (53%)	
Other*	35 (38%)	18 (33%)	17 (47%)	
Fast score (median [IQR])	6 [4, 7]	6 [5, 7]	5 [4, 6]	**<0.01**
Education				0.69
≤High School	9 (10%)	6 (11%)	3 (8%)	
> High School	82 (90%)	49 (89%)	38 (92%)	
Hispanic ethnicity				0.64
Non-Hispanic	71 (78%)	42 (76%)	29 (81%)	
Hispanic	20 (22%)	13 (24%)	7 (19%)	
Race				0.78
White	71 (78%)	42 (76%)	29 (81%)	
Black	13 (14%)	9 (16%)	4 (11%)	
Other race	7 (8%)	4 (7%)	3 (8%)	
B. Clinical factors (median [IQR])				
Social problem-solving skills				
Positive problem orientation	13 [10, 15]	13 [10, 15]	13 [10, 14]	0.67
Negative problem orientation	4 [2, 7]	5 [2, 7]	3 [1, 5.5]	0.16
Rational problem solving	10 [8, 12]	10 [8, 12]	10 [8, 12]	0.82
Impulsive/carelessness style	2 [1, 5]	2 [1, 4]	2.5 [1, 5]	0.66
Avoidance style	5 [3, 7]	5 [3, 7]	5 [3, 6]	0.18
Family caregiving (caregiving experience)				
Caregiver life satisfaction	20 [17, 24]	19 [17, 23]	22.5 [18, 25]	**0.05**
Caregiver social support	25 [23, 27]	26 [23, 27]	24 [23, 27]	0.39
Caregiver overload	11 [9, 12]	11 [9, 13]	10 [7, 12]	**0.05**
Satisfaction/love for caregiving role	27 [25, 30]	27 [25, 30]	26.5 [24, 30]	0.46
Resentment for caregiving role	14 [11, 17]	15 [11, 17]	14 [10.5, 16.5]	0.35
Anger toward care recipient	9 [6, 11]	9 [6, 11]	8.5 [6, 11]	0.44
Care recipient aggressive behaviors	11 [6, 15]	11 [8, 15]	10 [4.5, 15.5]	0.33
Care recipient depressive behaviors	6 [4, 8]	6 [5, 8]	7 [3, 8]	0.31
Care recipient forgetfulness/confusion	9 [8, 11]	9 [8, 11]	9 [8, 11]	0.41
Caregiver burden (ZBI)	37 [30, 46]	39 [32, 47]	32.5 [24, 42]	**0.01**
Depressive symptoms (PHQ-8)	6 [2, 8]	6 [3, 10]	3.5 [1, 6]	**<0.01**
Social disconnectedness (U-SIRS-13)	6 [3, 10]	7 [4, 10]	5 [2, 9]	0.07

^**±**^Responder = decrease (improvement) of 1 pt on ZBI equivalent to 1 SEM OR 3 + points on PHQ8. *Other diagnoses include Lew body dementia Vascular dementia Mild cognitive impairment and Mixed etiology and other Dementias.

*P*-values are bold when they are statistically significant at *p* <= 0.05.

We also assessed differences in Responders vs. Non-Responders for caregiver burden and for depressive symptoms separately. Participant characteristics and group comparisons for each outcome are presented in Supplementary Table 1 (Caregiver Burden Responders) and Supplementary Table 2 (Depressive Symptom Responders).

## Discussion

This study aimed to transcend outcome evaluation by examining the factors associated with treatment response among caregivers enrolled in a problem-solving intervention. We compared those achieving clinically significant improvement (i.e., Responders) with those who did not improve (i.e., Non-Responders) after PST/DSJ among 91 ADRD caregivers. Almost two-thirds of the sample were Responders (*n* = 55, 60.4%), supporting the benefits of PST/DSJ for dementia caregivers. Research on similar interventions has not always found statistically significant changes in burden/depression (55–57), and there is a paucity of dementia caregiver studies evaluating clinically, as opposed to just statistically, meaningful response to treatment (58).

In the current study, no personal characteristics of caregivers were associated with being a Responder (vs. Non-Responder). This suggests that the content of PST/DSJ was appropriate for caregivers in different situations and from different backgrounds and cultures, indicating success in our efforts to culturally translate the intervention while maintaining all evidence-based effective PST principles. Clinical factors associated with being a Responder (vs. Non-Responder) included providing care for individuals with greater dementia severity (moderately severe vs. midstage dementia) and, at baseline, reporting lower caregiver life satisfaction, higher caregiver overload, higher caregiver burden, and more depressive symptoms. This emphasizes that caregivers with greater need for intervention and support were also more likely to benefit from PST/DSJ.

This is contrary to a similar study in stroke caregivers who completed a cognitive-behavioral problem-solving and coping skills intervention that found that non-responders more often had a history of psychologic disorder and reported higher levels of anger than responders (19). Two notable differences between these studies may explain these somewhat discrepant findings. First, stroke and ADRD are notably different in their onset and progression, and participants in the stroke caregiver study were in their first year of caregiving. It may be that during this time of adjustment to a new, unexpected, and sudden-onset role, stroke caregivers experiencing the most emotional distress were not ready to engage in this kind of intervention. Second, related to the nature of the intervention, though both taught adaptive problem-solving based coping skills, cognitive behavioral approaches focus more on self-reflection and changing internal thoughts about a situation to improve emotional well-being, whereas PST/DSJ focuses only on the step-by-step applied process for goal setting and goal achievement known to have downstream effects on emotional health. Direct comparative studies of these different approaches may be warranted to best target interventions to caregivers’ needs and individual circumstances.

While the demand for caregiving interventions is growing alongside the rates of ADRD diagnoses and unpaid caregivers in the US (59, 60), the nature of caregiving demands, as well as feelings of burden and depression, may hinder caregivers from seeking out or participating in available and accessible interventions (61, 62). Additionally, although caregivers of care recipients with more severe dementia may be more likely to respond to intervention, the complexities of their caregiving situations and circumstances (and feelings of being overwhelmed and overburdened) may prevent them from engaging in multi-session interventions (63). The virtual delivery modality of PST/DSJ may overcome some of these barriers, as it is conducive to remotely reaching and serving caregivers with limited time and the inability to leave their care recipients alone while they travel to in-person interventions (39). Notably, though not statistically significant, a higher proportion of participants in the Responder group were employed (50% vs. 36%), lending support to the idea that those with the most demanding schedules may benefit the most from flexible and remotely delivered interventions.

This study is an important step to understanding differences between those who do and do not respond to interventions, which can provide insight into targeted recruitment strategies and adaptations for greater effectiveness. In the clinical trial from which this study was drawn (40), we employed brief screeners for caregiver burden and depression to ensure caregivers could potentially benefit from the intervention. As such, large proportions of caregivers engaged in the intervention had modifiable risk factors addressed by PST/DSJ, which may indicate why the intervention was generally successful across intervention doses and personal characteristics. While many studies have used this approach to recruit and engage appropriate participants (64–66), this strategy is recommended for future research and practice to avoid ‘floor effects’ (i.e., participants do not have risk at baseline and may not benefit from the intervention) and/or engaging participants with too high of risk at baseline, which may signal the need for advanced intervention with clinical professions (i.e., beyond the anticipated and feasible benefits participants can receive from the offered intervention) (67).

Given the smaller, yet considerable, proportion of caregivers categorized as Non-Responders to the intervention in the current study, there are clear opportunities to adapt or complement PST/DSJ to better meet the diverse and complex needs of caregivers. In these analyses, all participants across intervention arms were combined into a single group, thus not accounting for dose–response in the analyses. This is justified by the non-superiority effect of session number and booster sessions in a previous PST/DSJ publication (39); however, if all caregivers received the same dose, or if dose was more individually targeted to individual need, a larger proportion may have been Responders to the intervention. This may support future pragmatic trials that uniformly serve caregivers to assess clinical benefits in care burden and depression. While not all caregivers responded to the intervention in terms of caregiver burden and depression, other subjective benefits may have been obtained by these caregivers (and those who were responders to the intervention). Therefore, future research should assess other measures to document other benefits of PST/DSJ, such as relationship quality, perceived care quality, shared decision making, resilience, social connection, loneliness, and participants’ perceptions of benefit (68).

Finally, some among the Non-Responders may require additional or different intervention. PST/DSJ teaches a problem-solving strategy that provides a concrete adaptive coping skill for proactive problem solving and goal attainment. The downstream benefits of PST/DSJ on emotional outcomes like depressive symptoms likely result from enhance self-efficacy, goal attainment, and behavioral activation (55, 69–71). However, this may not be sufficient to address more severe depression that may require psychotherapy or pharmacological intervention. For caregiver burden, the skills learned in PST/DSJ help caregivers better manage their daily tasks and achieve goals that are important to them (55, 71), but this does not necessarily lessen the overall burden they still experience by the demands of caregiving and the potential lack of support available. Community-level interventions that provide tangible and instrumental support to caregivers are still needed to lessen these demands (14, 68).

### Limitations and future directions

Though baseline assessment of caregivers in this study was robust, multiple factors that could contribute to treatment response were not measured, including access to resources, socioeconomic factors, and social support. Our sample was somewhat homogenous with regard to demographics (predominantly White care partners) and geography, which may introduce bias and limit generalizability. Additionally, though PST/DSJ is offered in both English and Spanish, there were not enough Spanish-speakers to examine whether response differed by language of delivery, which is an important direction for future research. Though care recipients had several different dementia diagnoses, most had AD, and the amount and nature of caregiving help provided was not captured in detail. Lastly, as previously noted, we only measured two common outcomes for caregivers: depressive symptoms and caregiver burden. However, PST/DSJ does not target any specific outcome and may have conferred unmeasured benefits for both caregivers and their care recipients.

Future work is needed to identify other potential benefits and to further determine for whom PST/DSJ would be most beneficial. Participants in the Non-responder group reported less severe symptoms of caregiver burden and depression compared to Responders, suggesting perhaps these were not outcomes for which they needed intervention. However, dementia caregivers experience a range of other challenges, from social participation restrictions to loneliness to anger and resentment, to name a few. Follow-up qualitative studies could reveal outcomes that are most meaningful and could benefit from PST/DSJ. Some in the Non-responder group actually reported worsening symptoms, indicating a need for more targeted and/or intensive intervention to address these potentially more serious emotional symptoms. Additionally, there are other approaches to operationalizing “response” to intervention, such as the participant’s perspective of whether they improved or not, which may yield different results.

## Conclusion

This study is an important step in identifying the drivers of intervention response among caregivers of people living with ADRD. As seen in our study, most caregivers demonstrated a clinically meaningful improvement in caregiver burden and/or depressive symptoms after receiving PST/DSJ. Notably, those who responded to the intervention had higher symptoms of distress, caregiver burden, overload, and depressive symptoms; had lower life satisfaction; and had care recipients with more severe dementia. These results indicate that those benefiting may also be those most likely in need of this support. Additional studies are needed to drive adaptations and complementary support services to effective caregiver interventions to improve recruitment and increase impact.

## Data Availability

The raw data supporting the conclusions of this article will be made available by the authors, without undue reservation.
