# Supplementary material for: Problem-solving training to improve caregiver burden and depressive symptoms among dementia caregivers: personal and clinical factors of responders vs. non-responders
Source: Front Public Health. 2025 Oct 10;13:1682373. doi: 10.3389/fpubh.2025.1682373 (PMC12549259; doi:10.3389/fpubh.2025.1682373)
Supplement: Supplementary file 1 [file Supplementary_file_1.docx]

| **Supplemental Table 1. Differences in Personal and Clinical Factors Between Responders and Non-Responders to Problem-Solving Training for Caregiver Burden** | | | | |
| --- | --- | --- | --- | --- |
| **Personal Factor** | **All**  **N=91** | **Responders^±^**  **N=51** | **Non-Responders**  **N=40** | **P-value** |
| Age years (median [IQR]) | 61 [52, 72] | 59 [51, 71] | 64.5 [52.5, 73] | 0.39 |
| Gender  CIS-Female  CIS-Male | 77 (86%)  13 (14%) | 44 (86%)  7 (14%) | 33 (85%)  6 (15%) | 0.82 |
| Employment  Yes  No | 42 (46%)  49 (54%) | 28 (55%)  23 (45%) | 14 (35%)  26 (65%) | 0.06 |
| Diagnosis of Care Recipient  Alzheimer’s Disease  Other^*^ | 56 (62%)  35 (38%) | 34 (67%)  17 (33%) | 22 (55%)  18 (45%) | 0.26 |
| Fast Score (median [IQR]) | 6 [4, 7] | 6 [4, 7] | 5 [4, 6] | 0.12 |
| Education  <= High School  > High School | 9 (10%)  82 (90%) | 4 (8%)  47 (92%) | 5 (13%)  35 (88%) | 0.46 |
| Hispanic Ethnicity  Non-Hispanic  Hispanic | 71 (78%)  20 (22%) | 40 (78%)  11 (22%) | 31 (78%)  9 (22%) | 0.92 |
| Race  White  Black  Other Race | 71 (78%)  13 (14%)  7 (8%) | 40 (78%)  8 (16%)  3 (6%) | 31 (78%)  5 (13%)  4 (10%) | 0.72 |
| **Clinical Factors** (median [IQR]) | **All**  **N=98** | **Responders**  **N=58** | **Non-Responders**  **N=40** | **P-value** |
| Social Problem-Solving Skills  Positive Problem Orientation  Negative Problem Orientation  Rational Problem Solving  Impulsive/Carelessness style  Avoidance Style | 13 [10, 15]  4 [2, 7]  10 [8, 12]  2 [1, 5]  5 [3, 7] | 13 [10, 15]  5 [2, 7]  11 [9, 13]  2 [1, 4]  5 [3, 7] | 13 [9.5, 14]  3.5 [1, 5.5]  10 [8, 12]  3 [1, 5]  4.5 [3, 6] | 0.35  0.21  0.30  0.46  0.09 |
| Family Caregiving (Caregiving Experience)  Caregiver Life Satisfaction  Caregiver Social Support  Caregiver Overload  Satisfaction/Love for caregiving role  Resentment for caregiving role  Anger towards care recipient  Help Needed^¥^  Help Provided by Care Partner^¥^  Care recipient Aggressive behaviors  Care recipient Depressive behaviors  Care recipient Forgetfulness/Confusion | 20 [17, 24]  25 [23, 27]  11 [9, 12]  27 [25, 30]  14 [11, 17]  9 [6, 11]  6 [4, 14]  6 [4, 12]  11 [6, 15]  6 [4, 8]  9 [8, 11] | 19 [17, 23]  25 [23, 27]  11 [9, 12]  27 [25, 30]  15 [11, 18]  9 [6, 11]  8.5 [5, 15]  7.5 [4, 13]  11 [7, 15]  6 [5, 8]  9 [8, 11] | 22 [18, 25]  24 [23, 27]  10.5 [7, 12]  26.5 [24, 30]  14 [10.5, 16.5]  9 [6, 11]  4 [2, 11]  4 [2, 11]  10 [5, 15.5]  7 [3.5, 8.5]  9.5 [8, 11] | 0.12  0.58  0.13  0.29  0.24  0.58  0.06  0.14  0.64  0.77  0.99 |
| Caregiver Burden (ZBI) | 37 [30, 46] | 39 [32, 48] | 34.5 [24, 42.5] | **0.02** |
| Depressive Symptoms (PHQ-8) | 6 [2, 8] | 6 [3, 10] | 4 [1, 6] | **0.01** |
| Social Disconnectedness (U-SIRS-13) | 6 [3, 10] | 8 [5, 10] | 4 [2, 9] | **0.01** |
| **^±^** Responder=decrease (improvement) of 1 pt on ZBI equivalent to 1 SEM  ^*^Other diagnoses include Lew body dementia Vascular dementia Mild cognitive impairment and Mixed etiology and other Dementias  ^¥^Only asked if care partners indicated that the participant needed assistance hence the smaller sample size for these two scales. N=61, N=38, N=23 | | | | |

| **Supplemental Table 2. Differences in Personal and Clinical Characteristics Between Responders and Non-Responders to Problem-Solving Training for Depressive Symptoms** | | | | |
| --- | --- | --- | --- | --- |
| **Personal Factor** | **All**  **N=91** | **Responders^±^**  **N=27** | **Non-Responders**  **N=64** | **P-value** |
| Age years (median [IQR]) | 61 [52, 72] | 63 [54,70] | 60 [51.5, 72.5] | 0.46 |
| Gender  CIS-Female  CIS-Male | 77 (86%)  13 (14%) | 22 (81%)  5 (19%) | 55 (87%)  8 (13%) | 0.47 |
| Employment  Yes  No | 42 (46%)  49 (54%) | 12 (44%)  15 (56%) | 30 (47%)  34 (53%) | 0.83 |
| Diagnosis of Care Recipient  Alzheimer’s Disease  Other^*^ | 56 (62%)  35 (38%) | 17 (63%)  10 (37%) | 39 (61%)  25 (39%) | 0.86 |
| Fast Score (median [IQR]) | 6 [4, 7] | 6 [5, 7] | 6 [4, 6] | 0.17 |
| Education  <= High School  > High School | 9 (10%)  82 (90%) | 4 (15%)  23 (85%) | 5 (8%)  59 (92%) | 0.31 |
| Hispanic Ethnicity  Non-Hispanic  Hispanic | 71 (78%)  20 (22%) | 20 (74%)  7 (26%) | 51 (80%)  13 (20%) | 0.55 |
| Race  White  Black  Other Race | 71 (78%)  13 (14%)  7 (8%) | 20 (74%)  5 (19%)  2 (7%) | 51 (80%)  8 (13%)  5 (8%) | 0.75 |
| **Clinical Factors** (median [IQR]) | **All**  **N=98** | **Responders**  **N=34** | **Non-Responders**  **N=64** | **P-value** |
| Social Problem-Solving Skills  Positive Problem Orientation  Negative Problem Orientation  Rational Problem Solving  Impulsive/Carelessness style  Avoidance Style | 13 [10, 15]  4 [2, 7]  10 [8, 12]  2 [1, 5]  5 [3, 7] | 11 [9, 14]  6 [3, 8]  10 [8, 13]  2 [1, 4]  5 [3, 7] | 13 [11, 15]  3 [1, 6]  10 [8.5, 12]  2.5 [1, 5]  5 [3, 6] | 0.13  0.05  0.82  0.63  0.56 |
| Family Caregiving (Caregiving Experience)  Caregiver Life Satisfaction  Caregiver Social Support  Caregiver Overload  Satisfaction/Love for caregiving role  Resentment for caregiving role  Anger towards care recipient  Help Needed^¥^  Help Provided by Care Partner^¥^  Care recipient Aggressive behaviors  Care recipient Depressive behaviors  Care recipient Forgetfulness/Confusion | 20 [17, 24]  25 [23, 27]  11 [9, 12]  27 [25, 30]  14 [11, 17]  9 [6, 11]  6 [4, 14]  6 [4, 12]  11 [6, 15]  6 [4, 8]  9 [8, 11] | 18 [17, 20]  25 [23, 26]  12 [10, 13]  27 [26, 30]  14 [12, 18]  9 [6, 12]  9 [5, 16]  8 [4, 14]  10 [7, 16]  6 [6, 8]  9 [8, 12] | 21.5 [18, 24.5]  25 [23, 27]  10 [8, 12]  27 [24, 30]  14 [11, 17]  9 [6, 11]  6 [4, 12.5]  5.5 [3, 12]  11 [5.5, 15]  7 [4, 8]  10 [8, 11] | **0.02**  0.69  **0.04**  0.32  0.36  0.77  0.32  0.33  0.68  0.52  0.92 |
| Caregiver Burden (ZBI) | 37 [30, 46] | 39 [35, 46] | 36 [25, 46] | 0.09 |
| Depressive Symptoms (PHQ-8) | 6 [2, 8] | 8 [6, 12] | 4 [1.5, 6.5] | **<.01** |
| Social Disconnectedness (U-SIRS-13) | 6 [3, 10] | 8.5 [5, 11] | 6 [3, 9] | **0.03** |
| **^±^** Responder=decrease (improvement) of 3+ points on PHQ8  ^*^Other diagnoses include Lew body dementia Vascular dementia Mild cognitive impairment and Mixed etiology and other Dementias  ^¥^Only asked if care partners indicated that the participant needed assistance hence the smaller sample size for these two scales. N=61, N=21, N=40 | | | | |
